# Antibiotic Resistance Profiles and Genomic Analysis of Endophytic Bacteria Isolates from Wild Edible Fungi in Yunnan

**DOI:** 10.3390/microorganisms13020361

**Published:** 2025-02-07

**Authors:** Shuqiong Yi, Haiyan Wu, Yingting Lin, Xiaoyan Cha, Ying Shang

**Affiliations:** 1 Faculty of Food Science and Engineering, Kunming University of Science and Technology, Kunming 650500, China; yishugiong20222125011@stu.kust.edu.cn (S.Y.); 20212114038@stu.kust.edu.cn (Y.L.); 20212114039@stu.kust.edu.cn (X.C.); 2 The First People’s Hospital of Yunnan Province and the Affiliated Kunhua Hospital of Kunming University of Science and Technology, Kunming 650032, China; wuhaiyan@stu.kust.edu.cn

**Keywords:** wild edible fungi, endophytic bacteria, antibiotics resistance, foodborne pathogens, ARG, genome assembly

## Abstract

The use of antibiotics has led to the emergence of antibiotic resistance, posing significant challenges in the prevention, control, and treatment of microbial diseases, while threatening public health, the environment, and food safety. In this study, the antibiotic resistance phenotypes and genotypes of 56 endophytic bacteria isolates from three species of wild edible fungi in Yunnan were analyzed using the Kirby-Bauer disk diffusion method and PCR amplification. The results revealed that all isolates were sensitive to ofloxacin, but resistance was observed against 17 other antibiotics. Specifically, 55, 53, and 51 isolates exhibited resistance to amoxicillin, penicillin, and vancomycin, respectively. Antibiotic resistance gene (ARG) detection indicated that the sulfonamide *sul1* gene had the highest detection rate (53.57%). Excluding the ARG that was not detected, the lowest detection rates were the sulfonamide *sul2* and *sul3* genes, both at 1.79%. Among six tetracycline resistance genes, only *tetK* and *tetM* were detected. For β-lactam antibiotics, *blaTEM*, *blaVIM*, and *blaSHV* genes were present, while *blaOXA* was absent. In aminoglycoside resistance genes, *aadB* was not detected, while detection rates for *aac(3′)-IIa*, *acrB*, and *aadA1* were 3.57%, 1.79%, and 37.5%, respectively. The chloramphenicol *Cat* gene was detected at a rate of 14.29%, whereas *floR* was absent. For polypeptide resistance, *VanC* was detected at 3.57%, with *EmgrB* not detected. All three quinolone genes were detected, with detection rates of 8.92% for *GyrA*, 39.29% for *GyrB*, and 37.5% for *ParC*. Through phylogenetic analysis, 12 isolates that are closely related to ten common foodborne pathogenic bacteria were further selected for whole-genome sequencing and assembly. Gene annotations revealed that each isolate contained more than 15 ARGs and over 30 virulence factors. Notably, the detection rate of antibiotic resistance phenotypes was higher than that of genotypes, highlighting the importance of studying phenotypic antibiotic resistance that lacks identifiable ARGs. This study enriches the research on endophytes in wild edible fungi and provides new data for microbial ecology and antibiotic resistance research. It also offers critical insights for monitoring microbial antibiotic resistance in wild edible fungi and potentially other food sources, contributing to more effective strategies for ecological protection, sustainable agricultural development, and public health security.

## 1. Introduction

Plant endophytes are a group of microorganisms that live in plant tissues, interstitial tissues, and organs at a certain stage or throughout their life cycle. Studies on the diversity of plant endophytes have shown that these species mainly include endophytic fungi, endophytic bacteria, endophytic actinomycetes, and so on [1]. The main members of the plant microbial community are endophytes, which inhabit plant tissues. It has been found that plant endophytes are an integral part of plant microcosm, and most of them assist in plant growth through various pathways [2]. Many studies have revealed beneficial relationships between endophytes and their hosts, promoting plant growth through mechanisms such as nitrogen fixation, phosphorus solubilization, secretion of iron carriers, production of volatile organic substances, enhancement of nutrient uptake, and suppression of pathogenic fungi [3]. Simultaneously, the host plant provides a habitat for endophytes. As the application of toxic chemical pesticides and the production and long-term use of chemical fertilizers in agriculture has led to significant environmental pollution and plant pathogen resistance, research has found that most endophytes can colonize in the internal tissues of plants, and can reduce pests and diseases and promote plant growth, thus circumventing the negative impacts of chemical pesticides and providing an effective and environmentally friendly alternative [4]. In recent years, with the gradual expansion of various cross-disciplinary research areas and the deepening of research methods, advances in molecular techniques have simplified the isolation and characterization of some non-culturable species, and the understanding of endophyte diversity, function, metabolism, and genetics has continued to improve [5,6].

The era of antibiotics arrived with the discovery of penicillin, and an increasing number of antibiotics have been found and used in the treatment of clinical diseases, thus greatly promoting the development and progress of human health and society. The discovery of antibiotics is a great advancement in the history of world medicine and the beginning of another difficult global problem. Research and development of new antibiotics slowed down in the late 1970s, and no new antibiotics with novel structures appeared after 1984 [7]. The alarming rise of bacterial antibiotic resistance has resulted from the widespread and improper use, or even abuse, of antibiotics, with antibiotic residues detected in water, livestock, and food [8].

Bacterial resistance refers to the ability of bacteria to withstand antimicrobial agents [9]. The emergence of bacterial antibiotic resistance is an ancient and natural phenomenon. After penicillin [7], scientists identified resistance to other antibiotics in certain bacteria, such as *Streptococcus* [10] and *Mycobacterium tuberculosis* [11]. Bacteria continually exhibit resistance to newly discovered and applied antibiotics, leading to increasing rates of antibiotic resistance [12,13]. The existence of multiple antibiotic-resistant bacteria and super bacteria, such as methicillin-resistant *Staphylococcus aureus* [14], is an important factor for the increase in patient mortality. The relationship between humans, animals, and the environment facilitates the widespread dissemination of resistant bacteria and resistance genes (ARGs). In particular, food can effectively transfer and spread antibiotic resistance genes to human intestinal microbiota through consumption, allowing additional microorganisms to acquire antibiotic resistance. Consequently, food is an important carrier of ARGs for transmission [15,16]. The types of antimicrobial drugs used by food animals are almost the same as those used in human medicine [17]. In some countries, the amount of antibiotics used for food animals and pets surpasses that used for human medical treatment [18]. This situation broadens the path for ARG transmission. While foodborne pathogens are the primary focus of antibiotic resistance monitoring and research, nonpathogenic bacteria are abundant, widely distributed, and form the backbone of the microbial ecosystem. Given that ARGs in pathogenic bacteria are likely to be transmitted by nonpathogenic bacteria as their original carriers [19], understanding and monitoring the antibiotic resistance of both pathogenic and nonpathogenic bacteria is essential.

The province of Yunnan is abundant in wild edible fungi, which thrive in rich environments that also support a diverse range of endophytic bacteria. As an integral part of the ecosystem, the endophytic communities associated with these wild edible fungi play a crucial role in maintaining ecological balance and biodiversity. However, there are few reports on endophytic bacteria pathogenicity and their antibiotic resistance in wild edible fungi [20]. If these endophytes exhibit antibiotic resistance, they could pose a potential threat to human health. By investigating their resistance mechanisms, we can assess and monitor the safety of consuming wild edible fungi, thereby reducing the risk of foodborne diseases. Therefore, studying the antibiotic resistance of endophytic bacteria in wild edible fungi is necessary to provide a reference and basis for clinical antibiotic use. At present, the most commonly used method to study bacterial resistance is to investigate the molecular mechanism of ARGs after bacterial isolation through traditional culture methods. In this study, endophytic bacteria isolated from 3 kinds of wild edible fungi were tested for antibiotic resistance, and their phenotypes were observed and analyzed through an antibiotic sensitivity test. At the same time, the results of PCR were combined with the results of ARG annotation to analyze antibiotic resistance genotypes.

## 2. Materials and Methods

### 2.1. Materials

A total of 56 isolates were isolated from 8 groups of 3 wild edible fungi from Yunnan, namely *Lactarius deliciosus* (5 samples), *Russula virescens* (2 samples), and *Cantharellus cibarius* (1 sample). Numbered H1, H2, H3, H4, H5, Q1, Q2, and J, these samples were procured from different wild fungus stores in Yunnan Province.

The following antibiotics were used: penicillin 10 µg, amoxicillin 10 µg, ampicillin 10 µg, ticarcillin 30 µg, ceftazidime 30 µg, cefotaxime 10 µg, gentamicin 10 µg, streptomycin 10 µg, neomycin 30 µg, tetracycline 30 µg, minocycline 30 µg, doxycycline 30 µg, vancomycin 30 µg, compound sulfamethoxazole 25 µg, chloramphenicol 30 µg, ofloxacin 5 µg, ciprofloxacin 5 µg, and imipenem 30 µg. All antibiotic disks were purchased from Oxoid, UK.

The following instruments and equipment were utilized: electronic balance (JD200-3, Shenyang Longing Electronics Co., Ltd., Shenyang, China); high-speed desktop centrifuge (H318K, Hunan Kuchen Equipment Co., Ltd., Changsha, China); AB Simplify PCR instrument (Applied Biosystems Co., Ltd., Mumbai, India); Namedrop 2000 ultra-micro UV spectrophotometer (Thermo Fisher Scientific Inc., Waltham, MA, USA); Gel DOC ZR gel imaging system (Bio-Rad Laboratories, Thane, India).

### 2.2. Isolation of Endophytic Bacteria from Wild Edible Fungi

The soil was rinsed off the surface of the samples with tap water, and roots that had come into contact with the soil were removed with a sterile scalpel. Sterilization of samples was carried out on an ultra-clean bench as follows: the sample surface was first sterilized with 75% ethanol and rinsed with sterile water three times; the filter paper was dried and then soaked in 75% ethanol for 2 min, rinsed with sterile water three to five times, and then washed with 2.5% sodium hypoammonate for 3 min; the samples were rinsed with sterile water three to five times to ensure that any microbial residues on the surface were thoroughly washed away [21].

A 10 g sample was weighed from each of the sterilized samples. Two methods [22] were employed for the isolation of endophytes: (1) tissue isolation method: the epidermis of the samples was removed with a sterile scalpel, and the internal tissues were extracted; this tissue was cut into 0.5 cm square pieces and placed on LB agar plate medium (Hope Bio-Technology Co., Ltd., Qingdao, China) at three equidistant points from the center using sterile tubes; (2) grinding isolation method: place the 0.5 cm square piece block into a sterilized mortar, add quartz sand and sterile water, grind thoroughly, and let it stand for 3 min, then use a pipette to extract 100 μL of supernatant, and spread onto LB agar plate medium, finally, use the las cleaned samples of sterile water as a control, with three parallels for both the control and experimental groups.

Each plate was incubated at a constant temperature of 37 °C. After bacterial growth, compared with the control group, according to the morphology of the colony, a small amount of bacteria from the edge of individual colonies was taken using a sterile inoculation loop for isolation and purification. Finally, isolated bacteria were preserved in 30% glycerin (*v*/*v* = 1:1) at −80 °C. The sample numbers were as follows: Huanggu ripe H11, H12, H13… H21, H22, H23…; Qingtou bacteria Q11, Q12, Q13… Q21, Q22, Q23…; and chicken oil fungus J1, J2…

### 2.3. Identification of Isolates Using 16S rRNA Sequencing

A bacterial genome extraction kit (TaKaRa MiniBEST Bacteria Genomic DNA Extraction Kit Ver.3.0, Dalian, China) was utilized to extract the DNA of isolated bacterial strains. The concentration and purity of the bacterial DNA were assessed using NanoDrop 2000 (Thermo Fisher Scientific, Waltham, MA, USA)and electrophoresis. The samples with high-quality DNA were subjected to PCR amplification with the universal 16S rRNA bacterial primer (Table 1). PCR was performed by using an ABI SimpliAmp thermal cycler (Applied Biosystems) with a 25 µL reaction system, containing 2.5 µL of 10× PCR buffer (Mg^2+^ plus), 2 µL of dNTP mixture (2.5 mM), 1 μL of each primer (10 µM), 0.2 μL of rTaq DNA polymerase (5 U/µL) (TaKaRa, Biotechnology, Dalian, China), 2 µL of the DNA template, and 16.3 µL of ultrapure water. The 16S rRNA PCR amplification was carried out with the following program: initial denaturation at 94 °C for 2 min; 30 cycles of denaturation at 94 °C for 30 s, primer annealing at 54 °C for 30 s, and primer extension at 72 °C for 1 min; and followed by a final extension at 72 °C for 10 min.

The amplified products were analyzed on 2% agarose gel electrophoresis with ethidium bromide. The electrophoresis buffer was 1× TAE with the DL2000 marker (TsingKe, Beijing, China) used as the molecular weight standard. PCR products were sent to Shanghai Sangon Co., Ltd., Shanghai, China, for sequencing. Finally, the 16S rRNA sequences of the sequenced samples were uploaded to the NCBI database and compared using the BLAST tool. Moreover, isolates with a homology rate exceeding 99% were considered to belong to the same species.

### 2.4. Phylogenetic Relationships Between Isolates and Common Foodborne Pathogens

A phylogenetic tree was established by comparing the 16S rRNA sequences of bacteria isolated from wild edible fungi with those of 10 common foodborne pathogens. MEGA7 software (Ver.7.0) was used to build the phylogenetic tree, and isolates with close phylogenetic relationships to foodborne pathogens were selected. The selected bacterial isolates were activated in LB liquid medium, centrifuged at 12,000 r/min for 10 min, and sent to Genesky Biotechnologies Inc. (Shanghai, China) for whole genome sequencing, gene assembly, and bioinformation analysis, including ARGs and virulence factors.

The sequences of foodborne pathogens were obtained and downloaded from GenBank database as follows: *Bacillus cereus* (NCBI login No.: ABDM0200053.1), *Escherichia coli* O157: H7 (NZ_JHNG1000174.1), *Listeria monocytogenes* (AARY02000494.1), *Salmonella* (KF509913.1), *Shigella* (HM565981.1), *S. aureus* (JJEZ010000047.1), *Vibrio parahaemolyticus* (NZ_AOMN01000024.1), *Campylobacter jejuni* (NZ_KK365725.1), *Cronobacter sakazakii* (NZ_AJKT01000053.1), and *Vibrio vulnificus* (NZ_LBNN01000067.1).

### 2.5. Antibiotic Sensitivity Tests of 56 Isolates

The antibiotic resistance phenotypes of the isolates were tested using the Kirby–Bauer disk diffusion method of the Clinical and Laboratory Standards Institute (CLSI) [24]. The types and concentrations of antibiotic-sensitive disks used were mentioned in Section 2.1. The mediums with the antibiotic-sensitive disks were inverted and incubated at 37 °C for 18 h within 15 min. The diameter of the bacteriostatic circle was measured and evaluated according to the *Enterobacteriaceae* standard established by CLSI for each antibiotic susceptibility test. This standard categorizes results into sensitive, intermediate, and resistant [25].

### 2.6. ARGs Detections of 56 Isolates

We searched for suitable ARGs based on the information provided by the Antibiotic Resistance Gene Database (ARDB). Some primers for ARGs were designed using Vector NTI software (v11.5.2), while others were obtained from references (Table S1) [26,27,28,29,30,31,32,33]. All primers were synthesized by Shanghai SANGON Biotechnology Co., Ltd., Shanghai, China. The PCR reaction system was the same as used in Section 2.3. The PCR products were detected via 2% agarose gel electrophoresis and sent to Shanghai SANGON Biotech Co., Ltd. for sequencing.

## 3. Results

### 3.1. DNA Extraction and 16S rRNA Amplification of 56 Isolates

**Figure 1:**
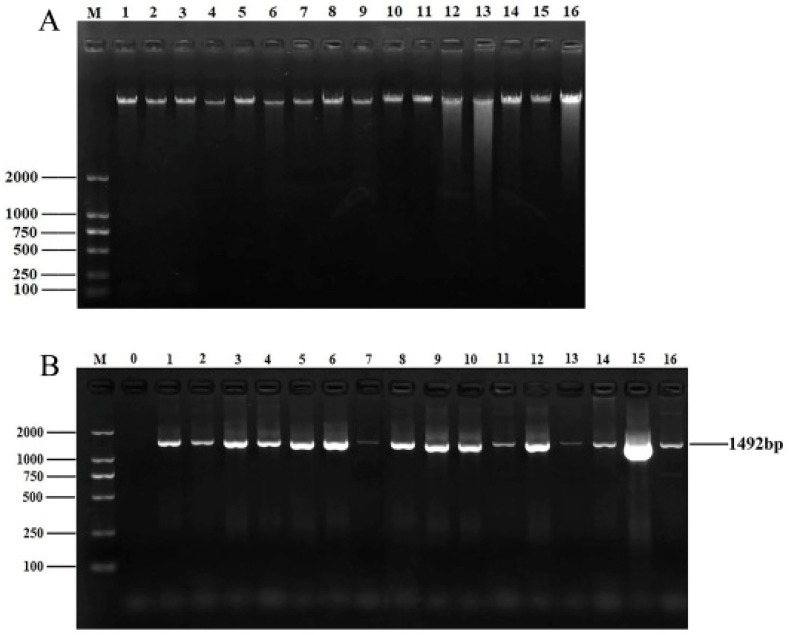
DNA extraction and 16S rRNA amplification of isolates. (**A**) Electrophoretic map of genomic DNA of partial isolates. M: DNA marker DL2000; lanes 1–16: genomic DNA of 16 isolates. (**B**) Electrophoretic map of 16S rRNA amplification products of partial isolates. M: DNA marker DL2000; lane 0: negative control; lanes 1–16: 16S rRNA amplification products of partial isolates.

After culture, isolation, and purification, a total of 56 endophytic bacterial isolates were obtained. DNA quality affects subsequent experiments. A bacterial genomic kit was used to extract and isolate the genomic DNA of the isolated strains. The concentration and purity of the strains were measured with an ultra–micro spectrophotometer. The OD260/280 ratios for all DNA ranged from 1.7 to 2.0, indicating that the purity of the extracted DNA was satisfactory. DNA quality was then analyzed via 1% agarose gel electrophoresis (Figure 1A). The DNA of all samples displayed clear and distinct bands, making it suitable for subsequent PCR amplification.

As shown in Figure 1B, all PCR bands of the isolates were clear and bright, with a length of approximately 1492 bp, which is consistent with the expected product size. After sequencing the PCR products, the 16S rRNA sequences of 56 isolates were uploaded to NCBI for BLAST comparison. Finally, these 56 bacterial isolates belonged to 14 genera, with *Pseudomonas*, *Cedecea*, *Serratia*, and *Lelliottia* being the dominant groups.

### 3.2. Phylogenetic Relationships Between Isolates and Common Foodborne Pathogens

**Figure 2:**
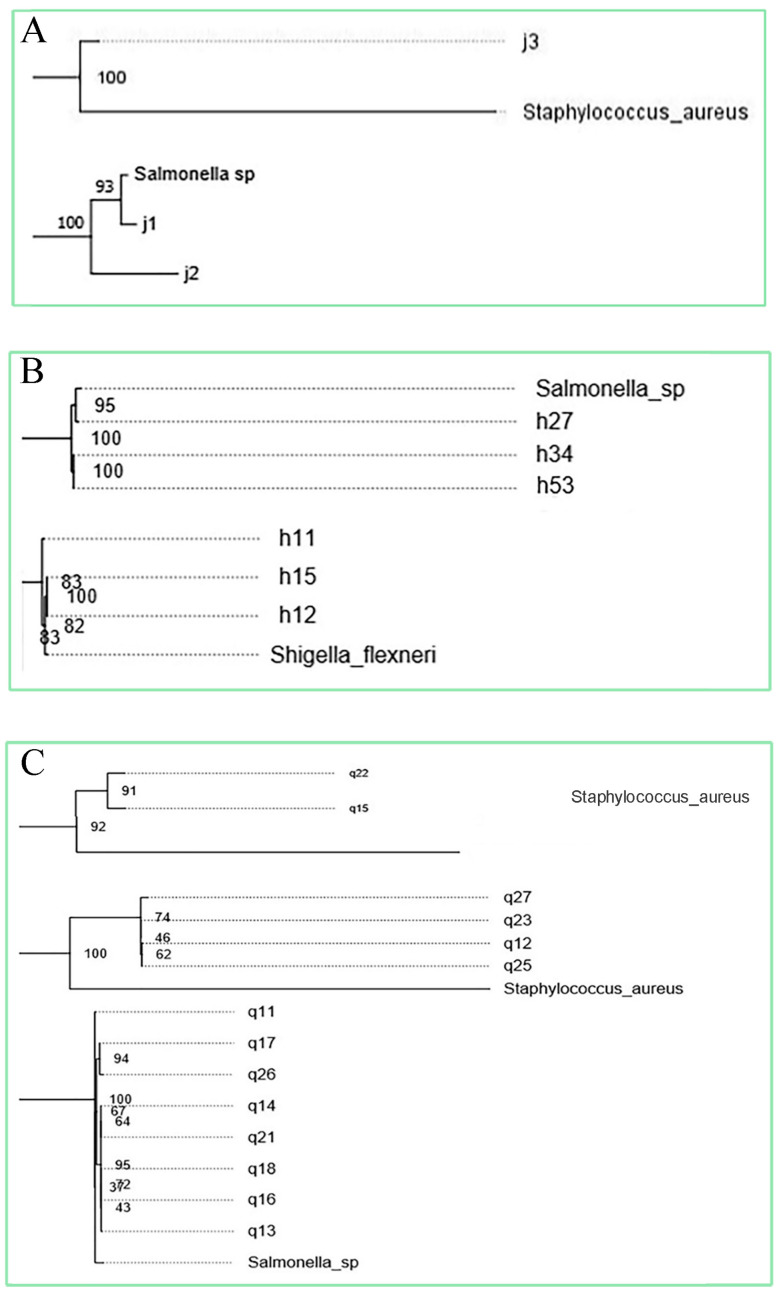
Phylogenetic tree among solates and common foodborne pathogens (**A**) Relationship between endophytic isolates of *Cantharellus cibarius* and foodborne pathogens. (**B**) Relationship between endophytic isolates of *Lactarius deliciosus* and foodborne pathogens. (**C**) Relationship between endophytic isolates of *Russul avirescens* and foodborne pathogens.

A phylogenetic tree was established between 56 isolates and the 10 subspecies of foodborne pathogens, and the relationship between them was determined. Isolates that have a phylogenetic relationship with foodborne pathogenic bacteria greater than 80 and have been reported in the reference as having pathogenic cases were selected. Finally, 12 strains were selected (Figure 2) and sent for whole genome sequencing, including J1, *Cedecea*; J2, *Enterococcus faecalis*; J3, *Cedecea*; H11, *Bacillus*; H15, *Pseudomonas*; H27, *Enterobacter*; H34, *Enterobacter*; H53, *Bacillus*; Q11, *Enterobacter*; Q15, *Serratia*; Q22, *Enterococcus*; Q23, *Cedecea*.

#### 3.2.1. Genome Assembly and Annotation

Trim Galore was used for quality assessment and filtering of the raw data, gene assembly was performed with SPAdes assembly software (4.0), and the GapCloser software (SOAPdenovo2-src-r240) was utilized to fill the local internal holes and correct the bases of the assembly results. The final assembly results are shown in Table S2. The assembly results were subjected to rRNA/tRNA gene prediction, Clusters of Orthologous Groups of proteins function analysis, and Kyoto Encyclopedia of Genes and Genomes pathway analysis.

#### 3.2.2. Virulence Factor Analysis

Based on the genome assembly and annotation, the predicted genes were compared with genes in the VFDB, an integrated and comprehensive database of virus factors for bacterial pathways, using blastp to identify the genes associated with virulence factors. VFDB contains two databases, setA and setB. SetA has been validated through experiments, making its results reliable. Consequently, we used the results of setA for virulence factor analysis. More than 30 kinds of virulence genes were found in the 12 isolates as shown in Table S3 (only 7 kinds of virulence factors represented a significant proportion). However, their mechanisms of action remain unclear and mainly include the formation of oligomer structure of hemolysin e; driven by an enzyme encoded by the iucbcd gene; binding with specific receptors; and other mechanisms. Additional mechanisms included antiphagocytosis, serum resistance, secretion system, effect on cell memory activity, and magnesium absorption.

#### 3.2.3. ARGs Analysis

The genes and mechanisms for antibiotic resistance were identified using blastp to compare predicted genes with those in the ARDB as shown in Table S4. The 12 isolates contained more than 15 kinds of ARGs, with the most prevalent resistance mechanisms being the efflux pump system and inactivated enzymes. Other resistance mechanisms included the resistance operon gene, superfamily transport, pentapeptide repeat family, and ribosome protection protein. These mechanisms can confer resistance to 15 kinds of antibiotics, including fosfomycin, macrolides, vancomycin, tetracycline, fluoroquinolone, penicillin, chloramphenicol, polymyxin, aminoglycoside, cephalosporin, *Bacillus subtilis* peptide, fosfomycin, and streptomycin.

### 3.3. Antibiotic Sensitivity Test Results of 56 Isolates

The diameter of the bacteriostatic circle did not exceed 1/3 of the culture dish. The edges of the bacteriostatic circle in the same culture medium with 3 different antibiotics-sensitive disks did not overlap. The bacteriostatic circles did not interpret each other or the interpretation results. Three disks were placed on 1 plate. After BLAST comparison of the 56 isolated, there was no accurate corresponding antimicrobial circle standard for the isolates in the CLSI standard. Therefore, the determination of the antimicrobial circle in this study was based on the CLSI standard for those of *Enterobacteriaceae*.

**Figure 3:**
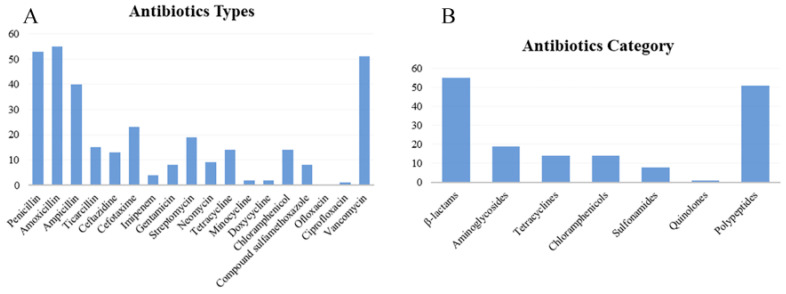
Distribution of antibiotics resistance phenotype of 56 isolates. (**A**) Antibiotics types; (**B**) antibiotics category.

In accordance with the criteria of the antimicrobial circle of *Enterobacteriaceae*, the resistance phenotypes of 56 isolates were statistically analyzed (Table S5, Figure 3). Table S5 and Figure 3A showed that among the 18 antibiotics, amoxicillin (98.21%), penicillin (94.64%), and vancomycin (91.07%) exhibited the highest resistance rates, while ofloxacin (0), ciprofloxacin (1.69%), minocycline (3.57%), and doxycycline (3.57%) had the lowest resistance rates. The highest sensitivity was observed with ofloxacin (100%), followed by ciprofloxacin (98.21%), and doxycycline (91.07%); the lowest sensitivity was recorded for amoxicillin (1.69%), followed by penicillin (5.36%) and vancomycin (8.93%). The highest intermediate antibiotic resistance rate was demonstrated by neomycin (30%), followed by streptomycin (16.07%) and imipenem (12.5%). The intermediate rates for sulfamethoxazole, ofloxacin, ciprofloxacin, and vancomycin were all 0. Figure 3B shows that 55 isolates were most resistant to β lactams, while 1 isolate exhibited the least resistance to quinolones. The category with the highest number of antibiotic-sensitive isolates is fluoroquinolone, with 56 isolates, while that with the fewest is the polypeptide, with 5 isolates. The highest number of isolates with intermediate-sensitive was 17, for aminoglycosides, and as to sulfonamides, quinolones, and polypeptides were 0. The strains that were sensitive to tetracycline were also sensitive to doxycycline and minocycline. More strains were sensitive to doxycycline and minocycline than to tetracycline. CLSI analysis showed that the isolates with intermediate resistance to tetracycline might be sensitive to doxycycline, minocycline, or both.

### 3.4. ARG Detection and Sequencing Comparison Results of 56 Isolates

**Figure 4:**
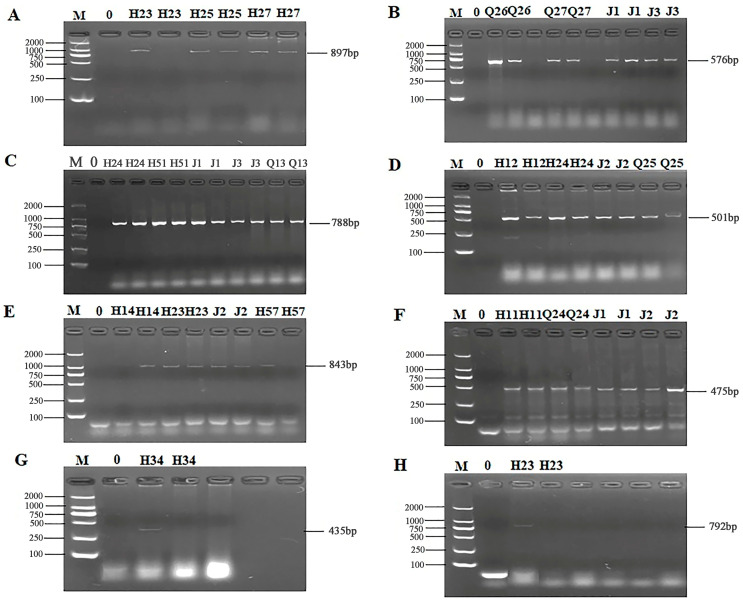
PCR results of some resistant genes. M: DL2000 marker; 0: negative control; (**A**) partial electrophoresis of *tetK* gene; (**B**) partial electrophoresis of *tetM* gene; (**C**) partial electrophoresis of *blaTEM* gene; (**D**) partial electrophoresis of *bla-vim* gene; (**E**) partial electrophoresis of *blaSHV* gene; (**F**) partial electrophoresis of *sul1* gene; (**G**) partial electrophoresis of *sul2* gene; (**H**) partial electrophoresis of *sul3* gene.

**Figure 5:**
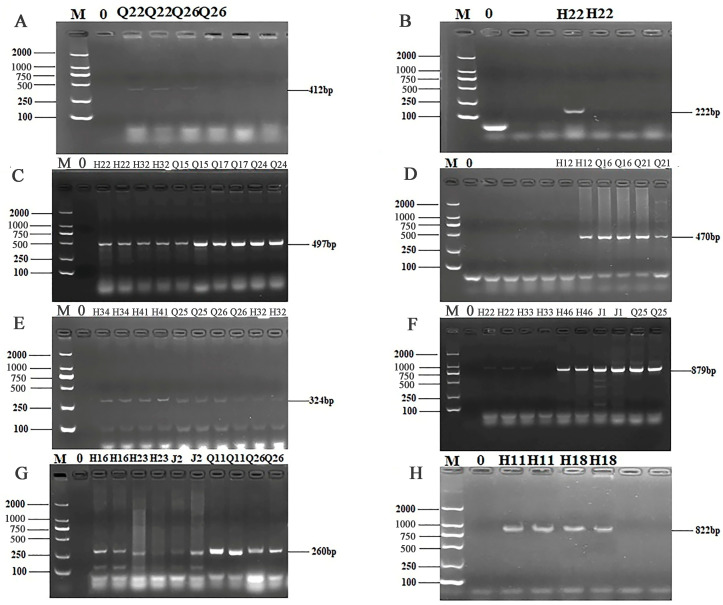
PCR results of some resistant genes. M: DL2000 marker; 0: negative control; (**A**) partial electrophoresis of *aac(3′)*—*IIa* gene; (**B**) partial electrophoresis of *acrB* gene; (**C**) partial electrophoresis of *aadA1* gene; (**D**) partial electrophoresis of *Cat* gene; (**E**) partial electrophoresis of *GyrA* gene; (**F**) partial electrophoresis of *GyrB* gene; (**G**) partial electrophoresis of *ParC* gene; (**H**) partial electrophoresis of *VanC* gene.

The 24 primers for ARGs were selected from 7 categories of antibiotics for PCR detection. The results of electrophoresis detection are shown in Figure 4 and Figure 5. Table S6 indicated that the highest detection rate was observed for the *sul1* (53.57%) gene in sulfonamides, while the lowest detection rate of 1.79% was found for the *sul2* and *sul3* genes, excluding isolates in which no PCR product was amplified. Among the 6 tetracycline resistance genes, only *tetK* (8.92%) and *tetM* (23.21%) were detected. The β-lactam genes *blaTEM*, *blavim*, and *blaSHV* were detected at the rates of 25%, 21.42%, and 23.21%, respectively. Among the 4 aminoglycoside resistance genes, *aadB* was not detected, while the detection rate for *aac (3′)-IIa*, *acrB*, and *aadA1* were 3.57%, 1.79%, and 37.5%, respectively. The detection rate of the chloramphenicol gene *Cat* was 14.29%, and *floR* was not detected. The peptide *VanC* gene was detected at the rate of 3.57%, and *EmgrB* was not detected. Three genes of quinolones, namely *GyrA* (8.92%), *GyrB* (39.29%), and *ParC* (37.5%), were detected. The PCR products also underwent sequencing, the sequences of gel-recovered PCR products were compared with the reference sequences in Genbank using blast, and the accession numbers of sequences with 99% similarity were recorded (Table S7).

### 3.5. Analysis of Multi-Antibiotics Resistance Spectrum

**Figure 6:**
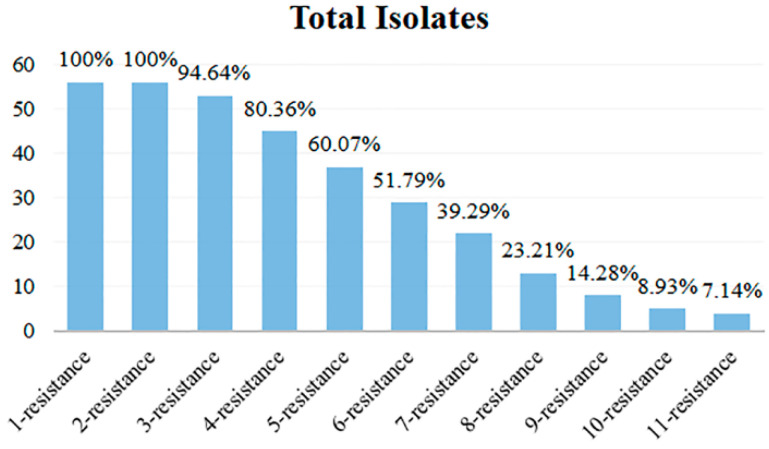
Multi-antibiotic resistance numbers of 56 isolates.

The antibiotic resistance spectrum of 56 isolates exhibiting various antibiotic resistance phenotypes was quantified, and the multi-antibiotic-resistant isolates and their types were analyzed. The results are shown in Table 2 and Figure 6. Among the 56 isolates, all isolates exhibited antibiotic resistance, with no cases of 0 resistance, while the others displayed a highly concentrated resistance profile ranging from 1 to 11. The most prevalent resistant types were 1-resistant and 2-resistant, with all 56 isolates exhibiting resistance, which accounted for 100%. Conversely, the isolates categorized as 11-resistance were the fewest, with only 4 isolates accounting for 7.14% of the total. Figure 6 illustrates that isolates with resistance to multiple antibiotics predominantly fell into the categories of 1- to 6-resistance, with their antibiotic resistance isolates exceeding 50%. The multi-antibiotic resistance spectrum with the most forms was 6-resistance, which encompassed 6 types. In contrast, the 3-, 9-, and 10-resistance had only 2 types. The 2-, 4-, and 11-resistance had 3 types, while 5- and 7-resistance exhibited 5 types, and the 8-resistance contained 4 types.

### 3.6. Analysis of the Coincidence Rate Between the Antibiotic Resistant Phenotype and Genotype of the 56 Isolates

The antibiotic resistance phenotypes and genotypes of the 56 isolates were compared, and the coincidence rate was analyzed (Table S8). The highest coincidence rate was 90.48% for aminoglycosides, while the lowest was 2.94% for quinolones. Antibiotics exhibiting more phenotypic isolates than genotypes included β-lactams, chloramphenicol, and peptides, whereas those with more genotypes than phenotypes included tetracyclines, sulfonamides, aminoglycosides, and quinolones.

### 3.7. Gene Annotation Result Matching of Antibiotic Resistance Gene Annotation of the 12 Isolates That Closely Related with the Foodborne Pathogens

As to the 12 isolates that closely related to the foodborne pathogens that were reported to be pathogenic, according to the antibiotic resistance genes annotation and antibiotic resistance genotype and phenotype comparison results (Table S9), no resistant genes for β-lactams or sulfonamides were found in the gene annotation. Additionally, the sulfonamide resistance genes *sul1* and *sul2*, as well as the aminoglycoside resistance gene *aadA1*, were not present in the resistance gene annotations. However, most other genes were all matched, with the same antibiotic resistance gene corresponding to multiple resistance mechanisms.

## 4. Discussion

There are various relationships between endophytic bacteria and their hosts, with the most researched being the positive effects they confer on the host plants, some even influence the quality of the host. The results of this study showed that endophytic bacteria isolated from wild edible fungi of the same host exhibit rich diversity. Furthermore, for wild edible fungi of the same species found in different locations, there are both similarities and differences among the endophytic bacteria. A total of 56 isolates were isolated and purified, identified through BLAST comparison, revealing 14 genera, with *Pseudomonas*, *Cedecea*, *Serratia*, and *Lelliottia* as the dominant bacterial groups. This demonstrates that the endophytes possess significant diversity, and their distribution and colony structure is influenced by the growth stage of the host plant and environmental conditions.

The resistance of bacteria to antibiotics is a common phenomenon in nature, but the abuse of antibiotics leads to bacterial tolerance. Consequently, the issue of acquired resistance to antibiotics, including new ones, is becoming increasingly serious. ARGs are transmitted both horizontally and vertically, resulting in the emergence of superbacteria resistant to multiple antibiotics [34,35]. In this study, 56 isolates of endophytic bacteria isolated from wild edible fungi were tested for antibiotic resistance. The antibiotic sensitivity test was performed with 18 antibiotics across 7 categories. The results indicated that all isolates were sensitive to ofloxacin, while the sensitivity rates for penicillin (the earliest used antibiotic for disease treatment), as well as the commonly used antibiotics amoxicillin and vancomycin, were 5.36%, 1.69%, and 8.93% respectively. The corresponding antibiotic resistance rates were 94.64%, 98.21%, and 91.07%. The resistance rate to ampicillin exceeded 70%; that to cefotaxime surpassed 40%; to streptomycin, it exceeded 30%; and to ticarcillin, ceftazidime, tetracycline, and chloramphenicol, they all exceeded 20%. Currently, carbapenems are effective in the treatment of Gram-negative bacterial infections [36] and are particularly effective against multidrug-resistant *Enterobacteriaceae* infections [37]. Imipenem is also a new carbapenem antibiotic, but bacteria have developed resistance to it due to its widespread clinical use. Monitoring network data on antibiotic-resistant bacteria in China indicated that the resistance rate of imipenem-resistant bacteria has gradually increased in recent years, a phenomenon that warrants attention. The endophytic bacteria examined in this study are sensitive to ofloxacin and exhibit varying degrees of resistance to 17 kinds of antibiotics. The prolonged misuse of antibiotics and the presence of antibiotic residues in food have impacted environmental microorganisms and the natural growth of plant tissue endophytes through the transfer of ARGs. The rise of antibiotic-resistant microorganisms has resulted in food safety and public health issues, further complicating clinical treatment.

In this study, 24 common ARGs were tested. The results indicated that 12 ARGs were detected among the 56 isolates. More phenotypes than genotypes were identified for β-lactams, chloramphenicol, and polypeptides, likely due to differing biochemical and genetic mechanisms of antibiotic resistance [38]. The β-lactams are the most commonly used antibiotics for treating bacterial infection. Their resistance mechanisms mainly involve the production of β-lactamases, efflux pump systems, and alterations in penicillin-binding proteins. The former can confer antibiotic resistance by hydrolyzing specific β-lactamases [39], such as metal β-lactamases and serine β-lactamases [40]. The mexab-OprM efflux pump system is the primary contributor to the inherent antibiotic resistance of bacteria [41] and enhances resistance against penicillin and cephalosporin. The chloramphenicol resistance mechanism mainly involves the active efflux system and the production of chloramphenicol acetyltransferase [42], with the acetyltransferase mechanism being the principal mechanism of chloramphenicol resistance [43].

Research on the mechanisms underlying vancomycin resistance in China has primarily focused on *Enterococcus* and *S. aureus*. Most *Enterococcus* bacteria develop resistance to vancomycin through the acquisition of ARGs and interspecific transmission [44,45]. The high level of resistance in *S. aureus* aligns with that of *Enterococcus*, while the low level of vancomycin resistance may result from multiple chromosomal site mutations [46] or regulation of protein expression [45]. Additionally, cell membranes and active efflux systems are prevalent in both Gram-negative and Gram-positive bacteria. Consequently, due to the biochemical mechanism of bacterial resistance, β lactams, chloramphenicol, and polypeptides exhibited more resistant genotypes than resistant phenotypes in this study. Furthermore, if the strain has an antibiotic-resistant phenotype but lacks the corresponding antibiotic-resistant genotype, it is likely inherently resistant to an antibiotic [47]. ARGs for tetracyclines, sulfonamides, aminoglycosides, and quinolones were detected, but few phenotypes were detected. ARGs may be present but silenced, not expressed, or not fully expressed. Qin et al. [26] conducted antibiotic resistance experiments on lactic acid bacteria isolated from commercial yogurt and found isolates carrying erythromycin and tetracycline ARGs but exhibiting sensitive phenotypes, demonstrating that no antibiotic resistance phenotype carried ARGs.

The analysis of the multi-antibiotics resistance spectrum among the 56 isolates reveals significant insights into the prevalence and distribution of antibiotic resistance phenotypes. The findings indicate that a substantial proportion of the strains exhibit high levels of resistance, particularly in the 1-resistance and 2-resistance categories, which accounted for all strains analyzed. This suggests a concerning trend in antibiotic resistance that could have implications for treatment strategies and public health. The concentration of isolates within the lower resistance categories (1 to 6) indicates that these isolates may be more adaptable or prevalent in the studied environment, potentially due to selective pressures from antibiotic use. The identification of only a few isolates in the higher resistance categories (such as 11-resistance) highlights the need for further investigation into the mechanisms driving such resistance and the factors contributing to the emergence of highly resistant strains. Moreover, the data suggest that multi-antibiotic resistance is a significant issue, with over 50% of strains exhibiting resistance across multiple antibiotic classes. This multi-antibiotic resistance complicates treatment options and underscores the urgency for developing new therapeutic strategies and implementing effective antibiotic stewardship programs.

Although foodborne pathogens are the primary focus of antibiotic resistance monitoring and research, nonpathogenic bacteria are abundant, widely distributed, and constitute the core of microbial ecosystems. Since the ARGs identified in pathogenic bacteria are likely transmitted by nonpathogenic bacteria as their original carriers [19], understanding and monitoring the antibiotic resistance of both pathogenic and nonpathogenic bacteria is crucial. Currently, a key index for assessing the safety of strains is the presence of ARGs. Research on the mechanism of antibiotic resistance primarily encompasses two aspects: biochemical and genetic mechanisms. In this study, the coincidence rates of antibiotic resistance phenotypes and genotypes were inconsistent. The main reason for these findings we speculated was the differing mechanisms of antibiotic resistance. Therefore, a thorough investigation into the resistance mechanisms of various isolates against different antibiotics, as well as exploring the reasons behind the prevalence of phenotype isolates over genotypes, is of great significance and provides valuable insights for studying antibiotic resistance and reducing antibiotic resistance rates.

## 5. Conclusions

In conclusion, the study highlights the alarming prevalence of multidrug-resistant strains among the analyzed samples. The findings demonstrate that all 56 strains exhibited some level of resistance, with a notable concentration in the lower resistance categories. The results emphasize the critical need for ongoing surveillance of antibiotic resistance patterns and the development of targeted interventions to combat the rise of multidrug-resistant infections. Addressing this issue is essential for ensuring effective treatment options and safeguarding public health.

## Figures and Tables

**Table 1:** 16S rRNA PCR universal primer amplification sequence.

Primers	Gene Sequences (5′→3′)	References
27-F	AGATTTGATCCTGGCTCAG	[23]
1492-R	CTACGGCTACCTTGTTACGA	

**Table 2:** Multi-antibiotic resistance types of 56 isolates.

Type	Antibiotics Resistance Spectrum	Isolates	Total Isolates	Rate
1-resistance	—	56	56	100%
2-resistance	AMX-CTX	23	56	100%
	AMX-CAZ	12		
	P-VA	21		
3-resistance	P-AMX-VA	51	53	94.64%
	AMX-CAZ-STX	2		
4-resistance	P-AMX-AMP-VA	20	45	80.36%
	P-AMX-N-VA	14		
	P-AMX-TE-N	11		
5-resistance	P-AMX-AMP-TE-VA	3	37	66.07%
	P-AMX-TE-N-VA	4		
	P-AMX-AMP-S-VA	18		
	P-AMX-AMP-VA-IPM	4		
	P-AMX-TE-S-VA	8		
6-resistance	P-AMX-AMP-TIC-CAZ-CTX	6	29	51.79%
	P-AMX-AMP-CTX-VA-STX	6		
	P-CAZ-S-N-TE-VA	2		
	P-AMX-TIC-CTX-VA-C	7		
	P-AMX-AMP-S-VA-C	3		
	P-AMX-CAZ-CTX-CN-VA	5		
7-resistance	P-AMX-AMP-CTX-S-VA-CIP	1	22	39.29%
	P-AMX-TIC-S-TE-VA-MN	2		
	P-AMX-AMP-TIC-TE-VA-C	9		
	P-AMX-CTX-CN-S-N-VA	6		
	P-AMX-S-TE-VA-MN-TIC	4		
8-resistance	P-AMX-AMP-CTX-S-VA-STX-TIC	4	13	23.21%
	P-AMX-AMP-CTX-CN-S-N-VA	4		
	P-AMX-AMP-S-TE-DO-VA-C	3		
	P-AMX-AMP-S-TE-DO-VA-SXT	2		
9-resistance	P-AMX-AMP-CTX-TIC-VA-STX-C-IPM	5	8	14.28%
	P-AMX-AMP-TIC-CAZ-CTX-S-VA-C	3		
10-resistance	P-AMX-AMP-CTX-CN-S-TE-VA-STX-C	3	5	8.93%
	P-AMX-AMP-CTX-IPM-S-TE-VA-STX-C	2		
11-resistance	P-AMX-AMP-TIC-CAZ-CTX-CN-N-S-VA-C	1	4	7.14%
	P-AMX-AMP-TIC-CTX-CN-S-N-TE-VA-C	2		
	P-AMX-AMP-TIC-CAZ-CTX-S-TE-VA-STX-C	2		

## Data Availability

The original contributions presented in this study are included in the article/Supplementary Materials. Further inquiries can be directed to the corresponding author.

## Supplementary Materials

The following supporting information can be downloaded at: https://www.mdpi.com/article/10.3390/microorganisms13020361/s1, Table S1: Primer sequences of drug resistance gene; Table S2: Assembly results; Table S3: Analysis of virulence factors; Table S4: Antibiotics resistance gene analysis; Table S5: Statistical results of antibiotics resistance phenotype of 56 strains; Table S6: Detection rate of drug resistance genes in 56 isolates; Table S7: Comparison of gel recovery and sequencing results; Table S8: Analysis of the coincidence rate between drug resistance phenotype and genotype; Table S9: Result matching of antibiotic resistance gene annotation of the 12 isolates that closely related with the foodborne pathogens.
