# Supplementary material for: Antibiotic Resistance Profiles and Genomic Analysis of Endophytic Bacteria Isolates from Wild Edible Fungi in Yunnan
Source: Microorganisms. 2025 Feb 7;13(2):361. doi: 10.3390/microorganisms13020361 (PMC11858796; doi:10.3390/microorganisms13020361)
Supplement: Supplementary file 1 [file microorganisms-13-00361-s001.zip › microorganisms-3400512-supplementary.pdf]

Table S1. Primer sequences of drug resistance gene.

Category	ARG	Sequence（5'→3'）	Annealing temperature（°C）	Target fragment（bp）	Reference
Tetracycline	*tetK*	F: TTAGGTGAAGGGTTAGGTCC<br>R: GCAAACTCATTCCAGAAGCA	59	897	[26]
	tetL	F: TCATCATCTCCTGATTTTAC<br>R: AGTAAAAACAAGCAGAGCAT	60	456	[27]
	tetM	F: GTTAAATAGTGTTCTTGGAG<br>R: CTAAGATATGGCTCTAACAA	55	576	
	tetO	F: AACTTAGGCATTCTGGCTCAC<br>R: TCCCACTGTTCCATATCGTCA	55	515	
	tetS	F: CATTTGGTCTTATTGGATCG<br>R: ATTACACTTCCGATTTCGG	55	573	[26]
	tetW	F: GAGAGCCTGCTATATGCCAGC<br>R: GGGCGTATCCACAATGTTAAC	58	168	
β-lac	*blaTEM*	F: CAGAAACGCTGGTGAAAG<br>R: TTACCAATGGTTAATCAGTGAG	56	788	[28]
	blavim	F: TTGGTCTACATGACCGCGTCTGTCA<br>R: AGATCGGCATCGGCCACGTT	59	501	[27]
	blaOXA	F: TTTTCTGTTGTTTGGGTTTC<br>R: TTTCTTGGCTTTTATGCTTG	55	447	
	blaSHV	F: TGTATTATCTCCCTGTTAGC<br>R: TTAGCGTTGCCAGTGCTC	57	843	[29]
Sulfonamides	*sul1*	F: TCGGACAGGGCGTCTAAG<br>R: GGGTATCGGAGCGTTTGC	63	475	
	sul2	F: CCTGTTTCGTCCGACACAGA<br>R: GAAGCGCAGCCGCAATTCAT	59	435	[27]
	sul3	F: ATGAGCAAGATTTTTGGAATCGTA<br>R: CTAACCTAGGGCTTTGGATATTT	57	792	[30]
Aminoglycosides	*aac（3'）-IIa*	F: GGCGACTTCACCGTTTCT<br>R: GGACCGATCACCCTACGAG	56	412	[29]
	acrB	F: CGTGAGCGTTGAGAAGTCCT<br>R: GGCGTCAGTTGGTATTTGGT	58	222	[31]
	aadB	F: GAGGAGTTGGACTATGGATT<br>R: CTTCATCGGCATAGTAAAA	55	208	[27]
	aadA1	F: TTTGCTGGTTACGGTGAC<br>R: GCTCCATTGCCCAGTCG	58	497	[30]
Chloramphenicol	*floR*	F: GAACACGACGCCCGCTAT<br>R: TTCCGCTTGGCCTATGAG	57	868	[29
	Cat	F: AGTGGAATAACGAACGAGC<br>R: TCAGCAAGCGATATACGCAG	57	470	[27]
Quinolones	*GyrA*	F: GGTGACGTAATCGGTAAATA<br>R: ACCATGGTGCAATGCCACCA	55	324	[29]
	GyrB	F: GGACAAAGAAGGCTACAGCA<br>R: CGTCGCGTTGTACTCAGATA	55	879	
	ParC	F: CTGGGTAAATACCATCCGCAC<br>R: CGGTTCATCTTCATTACGAA	55	260	
Polypeptides	*VanC*	F: GGTATCAAGGAAACCTC<br>R: CTTCCGCCATCATAGCT	50	822	[32]
	EmgrB	F: CCGCTGAGTAATAATCCTAT<br>R: TACAACCAAAGACGCAAT	60	492	[33]

Table S2. Assembly results.

Sample	Number of sequences (>= 0 bp)	Sum of sequence length (>= 0 bp)	Maximum contig length	GC (%)	N50 index	L50	Number of N /100 kbp
H11	6487	15662452	1074595	48	489910	10	1.35
H15	241	5164081	1385870	59.42	1383578	2	1.28
H27	154	5127050	1385882	59.44	1383477	2	0.49
H34	203	4984895	830172	53.87	317088	5	3.89
H53	967	10512185	570762	43.64	89260	30	1.81
J1	244	4784905	1622463	53.98	751508	2	4.65
J2	171	5356707	1652410	59.09	414925	3	0.06
J3	106	10015813	1022865	56.7	389997	8	0.07
Q11	187	4862404	2218828	55.96	520436	2	2
Q15	1210	5366476	2886859	59.53	2886859	1	1.21
Q22	161	5366476	1019797	59.11	414925	4	0.9
Q23	323	4793508	1573199	54.76	514850	3	0.06

Table S3. Analysis of virulence factors.

VF Name	Bacteria	Function Description	Function classification
LPS	*Bordetella pertussis*	Prevent host surfactant from clearing the body; protect bacteria from complement mediated cell lysis	endotoxin
Capsule	*Staphylococcus aureus*	Anti phagocytosis	Anti phagocytosis
TTSS(SPI-2 encode)	*Salmonella typhimurium*	Secretion of effector proteins to promote the replication of intracellular bacteria in membrane containing Salmonella vacuoles (SCV)	Type III secretion system
Alginate	*Pseudomonas aeruginosa*	Make bacteria form biofilm; contribute to the persistence of bacteria in CF lung: act as an adhesive to prevent bacteria from being discharged from the lung, sodium alginate mucus layer makes it more difficult for phagocytes to ingest and kill bacteria	Anti phagocytosis; serum resistance
Type IV pili	*Burkholderia pseudomallei*	Adhesion	Adhere
RicA	*Brucella*	Interaction with Rab2 in the form of GDP constraints: RicA-Rab2 interaction may affect the maturation of vacuoles containing Brucella in a way that slows down intracellular replication, thus avoiding the innate immune system	Cell memory activity
MgtBC	*Salmonella typhimurium*	MgtA and MgtB are not necessary for intracellular survival or virulence, but mgtc is necessary for both functions	Magnesium absorption

Table S4. Antibiotics resistance gene analysis.

Resistance Gene Type	Resistance Mechanism	Types of Antibiotic Resistance
rosb	Discharge pump / potassium transfer system. Rosa: Main accelerant superfamily transporter; Rasb: potassium transporter	Fosfomycin
macb	Antituberculosis cell division transporter system; multidrug resistance efflux pump; macrolides specific efflux system	Macrolides
vanre	VanE vancomycin resistant operon gene can modify D-Ala-D-Ala at the C-terminal to D-alanine-D-serine to synthesize peptidoglycan	vancomycin
tcma	The resistance of active tetracycline C efflux system to tetracycline C may be stimulated by transmembrane electrochemical gradient	tetracycline
qnrb	Pentapeptide repeat family, resistant to quinolones	Fluoroquinolone
pbp2	The enzyme has the N-terminal domain of penicillin insensitive trans glycosidase and the C-terminal domain of penicillin sensitive trans peptidase	penicillin
cml_e6	Main accelerant superfamily transporter; chloramphenicol efflux pump	chloramphenicol

arna	It catalyzes the oxidative decarboxylation of UDP GlcUA to udp-ara4o and adds formyl group to udp-l-ara4n to form udp-l-ara4fn bifunctional enzyme. The modified arabinose adheres to lipid A and is resistant to polymyxin and cationic antimicrobial peptide	Polymyxin
tetw	Ribosome protective protein	tetracycline
catb1	Group B chloramphenicol acetyltransferase, which can inactivate chloramphenicol, also known as exogenous acetyltransferase	chloramphenicol
emre	Multidrug resistant efflux pump	Aminoglycoside
bl1_sm	Class C β - lactamase, which destroys the β - lactamase antibiotic ring and makes the molecular antibacterial performance invalid	cephalosporin
bcra	ABC transport system; bacitracin efflux pump	Bacitracin
fosb	A metal glutathione transferase that confers fosfomycin resistance by catalyzing the addition of glutathione to fosfomycin	Fosfomycin
vatb	Virginia mycin A acetyltransferase can inactivate targeted drugs	streptomycin

Table S5. Statistical results of antibiotics resistance phenotype of 56 strains.

antibiotic	Sensitive Isolates	Intermediate Isolate	Resistant Isolates	Sensitivity Rate	Intermediary Rate	Resistance Rate
Penicillin	3	0	53	5.36% (3/56)	0（0/56）	94.64%（53/56）
Amoxicillin	1	0	55	1.69%（1/56）	0（0/56）	98.21%（55/56）
Ampicillin	11	5	40	19.64%（11/56）	8.93%（5/56）	71.43%（40/56）
Ticarcillin	36	5	15	64.29%（36/56）	8.93%（5/56）	26.78%（15/56）
Ceftazidine	39	4	13	69.64%（39/56）	7.14%（4/56）	23.22%（13/56）
Cefotaxime	31	2	23	55.36%（31/56）	3.57%（2/56）	41.07%（23/56）
Imipenem	45	7	4	80.36%（45/56）	12.5%（7/56）	7.14%（4/56）
Gentamicin	46	2	8	82.14%（46/56）	3.57%（2/56）	14.29%（8/56）
Streptomycin	28	9	19	50%（28/56）	16.07%（9/56）	33.93%（19/56）
Neomycin	30	17	9	53.57%（30/56）	30.36%（17/56）	16.07%（9/56）
Tetracycline	37	5	14	66.07%（37/56）	8.93%（5/56）	25%（14/56）
Minocycline	47	7	2	83.93%（47/56）	12.5%（7/56）	3.57%（2/56）
Doxycycline	51	3	2	91.07%（51/56）	5.36% (3/56)	3.57%（2/56）
Chloramphenicol	36	6	14	64.29%（36/56）	10.71%（6/56）	25%（14/56）
Compound sulfamethoxazole	48	0	8	85.71%（48/56）	0（0/56）	14.29%（8/56）
Ofloxacin	56	0	0	100%（56/56）	0（0/56）	0（0/56）
Ciprofloxacin	55	0	1	98.21%（55/56）	0（0/56）	1.69%（1/56）
Vancomycin	5	0	51	8.93%（5/56）	0（0/56）	91.07%（51/56）

Table S6. Detection rate of drug resistance genes in 56 isolates.

Category	Gene Name	Number of detected	Positive detection rate
Tetracyclines	*tetK*	5	8.92%
	tetL	0	0
	tetM	13	23.21%
	tetO	0	0
	tetS	0	0
	tetW	0	0
β-Lactams	*blaTEM*	14	25%
	blavim	12	21.42%
	blaOXA	0	0
	blaSHV	13	23.21%
Sulfonamides	*sul1*	30	53.57%
	sul2	1	1.79%
	sul3	1	1.79%
Aminoglycosides	*aac（3'）-IIa*	2	3.57%
	acrB	1	1.79%
	aadB	0	0
	aadA1	21	37.5%
Chloramphenicols	*floR*	0	0
	Cat	8	14.29%
Quinolones	*GyrA*	5	8.92%

	GyrB	22	39.29%
	ParC	21	37.5%
Polypeptides	*VanC*	2	3.57%
	EmgrB	0	0

Table S7. Comparison of gel recovery and sequencing results.

Gene Name	GenBank Login Number	Similarity
tetK	MN640712.1	100%
tetM	MH511633.1	100%
blaTEM	MN200750.1	100%
blavim	MN920417.1	100%
blaSHV	MN786391.1	100%
sul1	MH607135.1	100%
sul2	FJ670543.1	99.8%
sul3	MH765654.1	100%
aac(3')-IIa	MN069835.1	100%
acrB	MH933962.1	99.4%
aadA1	MN256779.1	100%
Cat	S48276.1	99.6
GyrA	MN076622.1	100%
GyrB	MK928264	100%
ParC	MG832641.1	99.7%
VanC	AF162694.1	99.7%

Table S8. Analysis of the coincidence rate between drug resistance phenotype and genotype.

Antibiotics Category	Phenotype Isolates	Genotype Isolates	Compliance Rate	
Tetracyclines	14	17	82.35%	(14/17)
Beta lactams	55	26	47.27%	(26/55)
Sulfonamides	8	30	26.67%	(8/30)
Aminoglycosides	19	21	90.48%	(19/21)
Chloramphenicols	14	8	57.14%	(8/14)
Quinolones	1	34	2.94%	(1/34)
Polypeptides	51	2	3.92%	(2/51)

Table S9. Result matching of antibiotic resistance gene annotation of the 12 isolates that closely related with the foodborne pathogens.

Types of Antibiotics	ARG Annotation	ARG PCR Amplication	Antibiotics-Resistant Mechanism
Tetracyclines	*tetT*、*tetO*、*tetX*、*tetH*、*tetM*、*tetK*、*tetV*、*tetC*、*tetpb*、*oprm*、*mexB*、*mexA*	*tetK*	Superfamily transporter; Tetracycline efflux pump
		tetM	Ribosome protection protein; Tetracycline efflux pump
β- lactams	*fosa*、*fosb*、*mdtg*、*mdth*	*blaTEM*	No match
		blavim	No match
		blaSHV	No match
Sulfonamides	*sul3*	*sul1*	No match
		sul2	No match
		sul3	Sulfadihydrowingerate synthase
Aminoglycosides	*emre*、*acrB*、*adeb*、*aac (3') -IIa*、*aac-6ie*、*acrA*	*aac(3')-IIa*	Aminoglycoside N-acetyltransferase
		acrB	Anti-tuberculosis cell division transporter system; multi-drug resistance efflux pump
		aadA1	No match

Chloramphenicols	*cm1*、*cat*、*ceoa*、*ceob*、*opcm*	*Cat*	Group A chloramphenicol acetyltransferase; Group B chloramphenicol acetyltransferase, also known as exogenous acetyltransferase
Quinolones	*mdtM*、*mdtK*、*norm*、*emea*、*oprn*、*mexf*	*GyrA*	No match
		GyrB	No match
		ParC	No match
Polypeptides	*arna*、*van*、*bcra*	*VanC*	Vancomycin resistance operon gene
